# Determinants and Time Trends for Ischaemic and Haemorrhagic Stroke in a Large Chinese Population

**DOI:** 10.1371/journal.pone.0163171

**Published:** 2016-09-29

**Authors:** Yutao Guo, Hao Wang, Tao Tao, Yingchun Tian, Yutang Wang, Yundai Chen, Gregory Y. H. Lip

**Affiliations:** 1 Department of Cardiology, Chinese PLA General Hospital, Beijing, China; 2 Department of Gerontology, Second People’s Hospital, Kunming, Yunnan Province, China; 3 University of Birmingham Centre for Cardiovascular Sciences, City Hospital, Birmingham, United Kingdom; University of Toronto, CANADA

## Abstract

**Background:**

The clinical epidemiology of stroke has been widely investigated in Caucasian populations, but the changes over time in the proportion of ischaemic to haemorrhagic strokes is less clear, especially in the Chinese population.

**Aims:**

Our objective was to study the determinants and time trends for ischaemic and haemorrhagic stroke, in relation to age, in a large Chinese population cohort.

**Methods:**

Using a medical insurance database in the southwest of China from 2001 to 2012, time trends in age-adjusted ischaemic and haemorrhagic stroke incidence and the contributing risk factors associated with age were investigated.

**Results:**

Among 425,901 individuals without prior stroke (52.4% male, median age 54), the rate of ischaemic stroke (per 1000 patient-years) decreased between 2002–2007, then remained broadly similar between 2008–2012. The rate of haemorrhagic stroke showed a similar trend, being approximately 1.3–1.9 from 2008–2012. Compared to patients age<65, ischaemic and haemorrhagic stroke incidences (rate, 95% confidential interval, CI) were higher in the elderly population (age <65 versus age ≥65: ischaemic: 3.64, 3.33–4.00, vs 14.33, 14.01–14.60; haemorrhagic: 1.09, 1.00–1.10 vs 2.52,2.40–2.70, respectively, both p<0.001). There were no significant differences in haemorrhagic stroke rates between the elderly and the very elderly population. Ischaemic and haemorrhagic stroke shared similar risk factors (age, hypertension, coronary artery disease (CAD), vascular disease, and diabetes mellitus) (all p<0.05). In subjects age<75 years, CAD (7.17, 4.14–12.37) and diabetes mellitus (3.27, 2.42–4.42) contributed most to the developing of haemorrhagic stroke (all p<0.001). Amongst the very elderly, vascular disease (2.24, 1.49–3.37) was an additional major risk factor for haemorrhagic stroke, together with CAD and diabetes mellitus (all p<0.001).

**Conclusion:**

In this large Chinese cohort, there was an increased risk of ischaemic stroke compared to haemorrhagic stroke with ageing. CAD, vascular disease, diabetes mellitus, and hypertension were major contributors to the development of hemorrhagic stroke in the very elderly Chinese population.

## Introduction

The prevalence, incidence, and life-time risk of ischaemic stroke have been investigated widely [1], and major risk factors (i.e. atrial fibrillation, AF) for ischaemic stroke have been identified [2]. Of these, there is a cumulative impact of multiple risk factors for ischaemic stroke risk [3,4, 5].

Much interest has been focused on ischemic stroke but amongst Asian subjects, there is a greater risk of haemorrhagic stroke (i.e. intracerebral hemorrhage (ICH), subarachnoid hemorrhage (SAH)) compared to non-Asians [6]. Of all strokes, ICH accounts for 10% and is associated with major disability and higher fatality [1], leading to a greater global burden. Haemorrhagic stroke incidence and mortality are significantly greater in developing countries compared to developed countries [7,8].

Overall stroke incidence in low-income and middle-income countries exceeds that seen in high-income countries by 20% in the 21st century [9]. The burden of stroke is particularly serious in Asia, and stroke mortality in Asia is even higher than in Europe or North America [10]. On the other hand, stroke incidence rates have fallen by 42% in high-income countries over past 4 decades, as has the stroke mortality [2]. This fall may be helped by antithrombotic therapy for ischaemic stroke prevention, based on the net clinical benefit balancing ischaemic and haemorrhagic events. This balance would be difficult in the elderly, who are at high risk for both ischaemic and haemorrhagic stroke. However, Asian subjects have a significant increase in ICH incidence compared to the Caucasian population [11], with a four-fold increased risk for ICH whilst on warfarin therapy [6].

The clinical epidemiology of stroke has been widely investigated in Caucasian populations, but the changes over time in the proportion of ischaemic to haemorrhagic strokes is less clear, especially in developing countries from Asia [12]. Our objective was to study the determinants, time trends and relation to age, for first-ever ischaemic and haemorrhagic stroke, in a large Chinese population cohort over a 10-year observational period.

## Methods

Databases used in this study have previously been described in detail [13]. The Chinese National Health Insurance program, which includes the Chinese medical insurance scheme, and Rural Cooperative Medical System, provide the basic medical care to urban and rural residents. The Chinese medical insurance scheme was started in December, 1998, and this program provides coverage for inpatient and outpatient medical services to approximately 597 million Chinese urban residents in 2014 (and covers 95% of total urban and rural residents in China). The local government maintains identical electronic clinical information on all health care provided to insured patients from the different provinces in China. In brief, we used the medical insurance databases affiliated with the Chinese medical insurance scheme in Yunnan Province, China, from January 1, 2001 through December 30, 2012.

The certified validated records provided by the hospitals were included into this governmental medical insurance claims database. Every individual participating in the medical health plan has a permanent and personal registration number, through which every medical ‘event’ could be identified, no matter whether the events happened in clinics and hospitals, and this would be written in the electronic medical records. Data captured included information on demography, diagnosis and treatment of various medical conditions.

### Sampling method

Sampling method was reported in our previous study [3]. All subjects were continually entered into the governmental medical insurance plan since 2001. The medical insurance data was compiled in Oracle RDBMS, version 10g (Oracle Corporation, Redwood Shores, California, USA). Structured Query Language (SQL) and systematic sampling using randomization blocks enabled random sampling of the study population. To avoid the less comprehensive coverage in the first several years of this Chinese medical insurance project, a sampling strategy according to year strata was taken. A random five-percent sampling was performed among the enrolled individuals biennially, according to 2001–2002, 2003–2004, 2005–2006, 2007–2008, 2009–2010, 2011–2012 to make the data representing the population into the medical insurance project every two years. Thus, a total of 1,228,639 persons were selected, but after excluding persons with incomplete data (n = 2611) and readmissions (n = 754,582), we identified 471,446 cases for analysis. Of this cohort, 425,901 without history of stroke were identified. The incidences of ischaemic or haemorrhagic stroke were studied from 2002 to 2012. Over the total follow-up of 1,895,447 person-years, there were 13274 incident ischaemic strokes and 2917 incident haemorrhagic strokes for the final analysis (S1 Fig).

The Medical Ethics Committee of PLA General Hospital has been approved by the China Food and Drug Administration (CFDA) (Registry number: XZF20120145) and this ethics committee approved the present study protocol (Approval number: 13BJZ40). The patient records/information was anonymized and de-identified prior to analysis. The database in this study was held by the government of Yunnan Province, China, which was managed with Center for Medical Insurance, Human Resources and Social Security, Yunnan Province (http://www.ynhrss.gov.cn/index.html).

### Evaluation of ischaemic stroke, haemorrhagic stroke and comorbidities

Ischaemic and haemorrhagic stroke (intracerebral and subarachnoid haemorrhage) were defined as “a focal or global neurologic deficit of sudden onset, developing clinical symptoms and/or signs, loss of cerebral function, with symptoms lasting more than 24 hours or leading to death”, diagnosed clinically by a neurologist and confirmed by CT or MRI. Detailed clinical information on ischaemic stroke, haemorrhagic stroke, and associated comorbidities were based on ICD-9 and ICD-10 codes. The index date was the first date of diagnosis of ischaemic or haemorrhagic stroke.

Ischaemic stroke cases were identified by International Classification of Disease, 9th Revision [ICD-9] or International Classification of Disease, 10th Revision [ICD-10] codes 436 or I63. Haemorrhagic stroke cases were identified by ICD-9 or ICD-10 codes 430,431,432; I60.x, I61.x. Heart failure(ICD-9 codes:428; ICD-10 codes: I42, I50, I110,J819), dilated cardiomyopathy (ICD-9 codes:425.4; ICD-10 codes: I42.0), diabetes (ICD-9 codes:249–250; ICD-10 codes: E10-E14), hypertension(ICD-9 codes:401–405; ICD-10 codes: I10-I15), coronary artery disease (ICD-9 codes:410–414; ICD-10 codes: I20-I25), myocardial infarction(ICD-9 codes:410; ICD-10 codes: I21,I22), peripheral vascular disease(ICD-9 codes:440.2; ICD-10 codes: I65, I70-74), chronic obstructive pulmonary disease(ICD-9 codes:490–496; ICD-10 codes: J42,J44.0–9), hyperlipidemia (ICD-9 codes:272.4; ICD-10 codes: E78.0–3,E78.5), renal dysfunction(ICD-9 codes:585,586; ICD-10 codes: M1A.3), hyperthyroidism(ICD-9 codes:242; ICD-10 codes: E05), hypothyroidism(ICD-9 codes:244; ICD-10 codes: E03), rheumatic heart disease (ICD-9 codes:393–398; ICD-10 codes: I05,I06,I07,I09.9). ICD-9, ICD-10 codes defined cardiovascular disease and other comorbidities are shown in S1 Table. The definition of various comorbidities is summarized in S2 Table.

Data for first hospitalization for ischaemic and haemorrhagic strokes from 2002 to 2012 year were retrieved from the medical insurance databases. This study did not include data for outpatients.

### Statistical analysis

Continuous variables were tested for normality by the Kolmogorov-Smirnov test. Those with a normal distribution are presented as a mean (standard deviation, SD) and analyzed using t test. Data with a non-normal distribution are presented as median (inter-quartile range, IQR). The comparison of discrete variables was performed using the chi-square test.

Incident ischaemic and haemorrhagic strokes (per 1000 person-years, 95% confidential interval, CI) were calculated in this population during a 10-year period. The rates of ischaemic and haemorrhagic stroke were calculated in relation to different age group categories (age <65 years, age 65–74 years, and age ≥75 years), and the relative rate ratio for ischaemic to haemorrhagic stroke in the three age groups over time were compared.

A multivariate analysis was used to determine cardiovascular risk factors predicting the occurrence of ischaemic and haemorrhagic stroke in the general populations, respectively. Factors associated with stroke were included into the Cox hazard proportional models, including age≥65, sex, CAD, vascular disease, hypertension, diabetes mellitus, atrial fibrillation (AF), heart failure(HF), and renal dysfunction. Hazard ratios (HR) of cardiovascular risk factors for ischaemic and haemorrhagic stroke were estimated by a Cox proportional hazards model. A p value <0.05 was considered as statistically significant. The 95% confidential interval (CI) were calculated based on Poisson distribution. Statistical analysis was performed using IBM SPSS Statistics version 21.0 (SPSS, Inc., Chicago, Illinois).

## Results

### Ischaemic strokes in relation to age, between 2002–2012

Among 425,901 individuals without prior stroke (52.4% male, median age 54), there were 13274 (63.8% male, median age 69) incident ischaemic strokes between 2002 to 2012. Hypertension, diabetes mellitus and CAD were the most comorbidities (Table 1).

**Table 1:** Demographic data of subjects with ischaemic and haemorrhagic stroke

Characteristics	Ischaemic stroke	Haemorrhagic stroke	p
n	13274	2917	
Age< 65 years, n(%)	4738(35.7%)	1417(48.6%)	**<0.001**
Age 65 to 74 years, n(%)	4371(32.9%)	797 (27.3%)	**<0.001**
Age ≥75 years, n(%)	4099(30.9%)	690(24.1%)	**<0.001**
Male, n(%)	8470(63.8%)	1977(67.8%)	**<0.001**
Hypertension, n(%)	2717(20.5%)	438(15.0%)	**<0.001**
Diabetes mellitus, n(%)	562(4.2%)	50(1.7%)	**<0.001**
Coronary heart disease, n(%)	391(2.9%)	20(0.7%)	**<0.001**
Hyperlipidemia, n(%)	375(2.8%)	33(1.1%)	**<0.001**
Vascular disease, n(%)	315(2.4%)	85(2.9%)	**<0.001**
Chronic kidney disease, n(%)	46 (0.3%)	6(0.2%)	0.163
Atrial fibrillation, n(%)	58(0.4%)	2(0.1%)	**0.003**
Congestive heart failure	25(0.2%)	4(0.1%)	0.607
*COPD, n(%)	18(0.1%)	1(0.0%)	0.230
Aspirin, n(%)	5456(41.1%)	147(5.0%)	**<0.001**
Clopidogrel, n(%)	357(2.7%)	10(0.3%)	**<0.001**
Warfarin, n(%)	32(0.2%)	—	-
*ACEI/ARB, n(%)	2571(19.4%)	641(22.0%)	**<0.001**
B blocker, n(%)	1353(10.2%)	270(9.3%)	**0.024**
Statin, n(%)	3407(25.7%)	187(6.4%)	**<0.001**
Digoxin, n(%)	78(0.6%)	8 (0.3%)	0.074
Diuretic, n(%)	1197(9.0%)	586(20.1%)	**<0.001**
Calcium antagonist, n(%)	5285(39.8%)	1084(37.2%)	**<0.001**
Nitrate, n(%)	290(2.2%)	197(6.8%)	**<0.001**

* COPD: Chronic obstructive pulmonary disease, ACE: Angiotensin converting enzyme; ARB: Angiotensin receptor blocker. The information of drug use was collected on discharge.

Ischaemic stroke rates (per 1000 patient-years, 95% CI) were 6.99 (6.90–7.10) during the 10-year period. The rate of ischaemic stroke decreased between 2002–2007, then remained broadly similar between 2008–2012.

**Fig 1:**
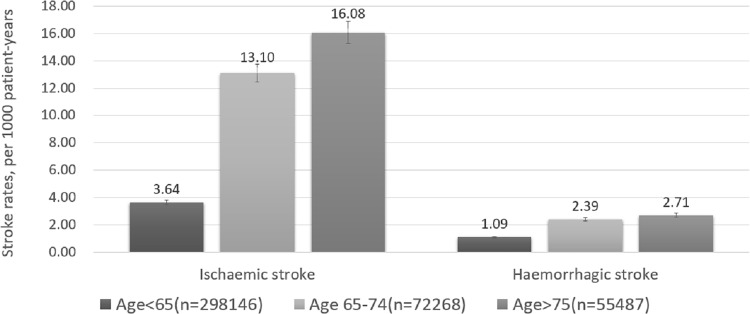
Rates of ischaemic stroke and hemorrhagic stroke in different age group.

**Fig 2:**
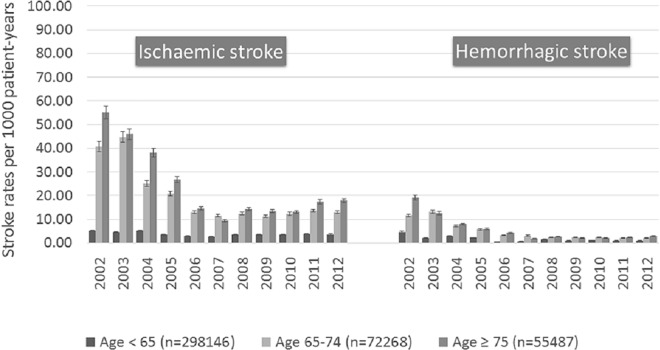
Time trends of ischaemic and hemorrhagic stroke incidences in different age groups.

The population with ischaemic stroke in 2012 was on average 7 years younger than those in 2002 (mean age, SD: 67.7, 11.1; vs 74.9, 9.4) (p<0.05). Compared to patients age<65, ischaemic stroke incidences were higher in the elderly (age <65 years versus age ≥65 years: 3.64 (3.33–4.00) and 14.33 (14.01–14.60), respectively, p<0.001) (Fig 1). When categorized as age <65 years, age 65–74 and age ≥75, ischaemic stroke incidences increased significantly in the very elderly population during this 10-year period (p<0.001) (Fig 2).

### Haemorrhagic strokes in relation to age, between 2002–2012

There were 2917 incident haemorrhagic strokes (67.8% male, median age 65) between 2002 to 2012, while the hypertension, vascular disease, and diabetes mellitus were the most comorbidities (Table 1).

**Fig 3:**
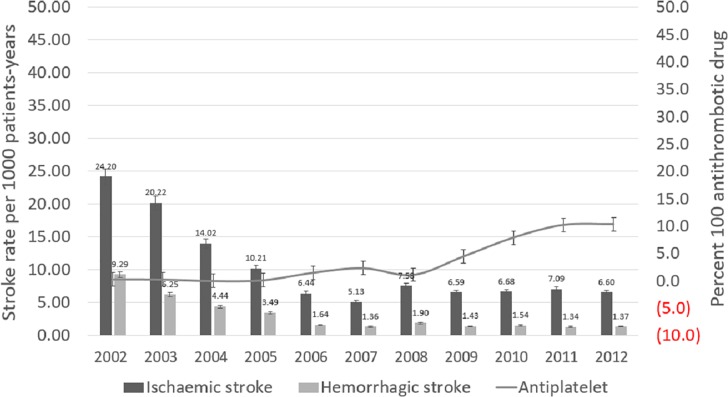
Rates of ischaemic stroke and hemorrhagic stroke in 425901 Chinese from 2002 to 2012 year. Ischaemic stroke: n = 13274; Hemorrhagic stroke: n = 2917.

Haemorrhagic stroke rates (per 1000 patient-years, 95% CI) were 1.53 (1.50–1.60) within the 10-year observational period. The rates of haemorrhagic stroke showed a decreasing trend between 2002–2007, and remained approximately 1.3–1.9 from 2008–2012 (Fig 3). There was a rise in antiplatelet therapy use between 2008 to 2012, ranged from 2.4% to 10.4% (Fig 3).

The population with haemorrhagic stroke was on average 8 years younger in 2012, compared to 2002 (mean age, SD: 62.8, 13.3; vs 71.0, 9.3) (both p<0.05). Compared to patients age<65, haemorrhagic stroke incidences were higher in the elderly population (age <65 years vs age ≥65 years: 1.09(1.00–1.10) and 2.52(2.40–2.70), respectively, p<0.001). There was no significant difference in haemorrhagic stroke between the elderly and the very elderly population (age 65–74 years vs age ≥75 years: 2.39 (2.21–2.60), 2.71(2.50–2.92), p = NS) (Fig 1). A similar non-significant trend for haemorrhagic stroke between the elderly and the very elderly population was seen throughout 10-year period (Fig 2).

The relative rate ratio of ischaemic to haemorrhagic stroke was 3.3 (3.64/1.09) in the population age <65 years, increasing to 5.48 (13.10/2.39) and 5.93(16.08/2.71) in age 65–74 and age ≥75 categories, respectively (p<0.001) (Fig 1).

### Drug therapies in relation to age

**Fig 4:**
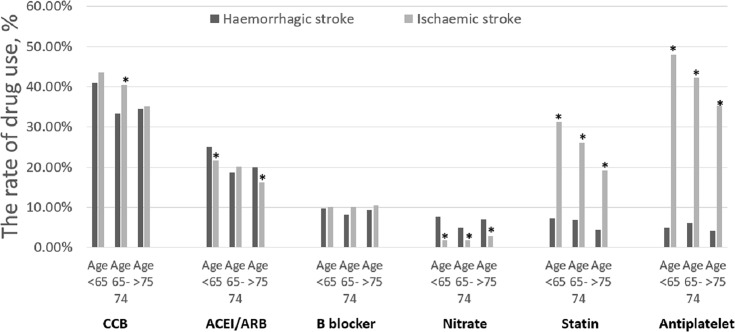
Drug therapies in patients with ischaemic and haemorrhagic stroke in relation to age groups. * Comparisons within the same age category, p<0.05.

Compared to the population with haemorrhagic stroke, the population with ischaemic stroke received more statins and antiplatelet therapy in all three age groups (all p <0.001) (Fig 4). With ageing, the use of CCB, ACEI/ARB, statin and antiplatelet in population with ischaemic stroke decreased (p for trend, <0.001), however, the trend was less evident with haemorrhagic stroke (p for trend, NS) (Fig 4).

### Multivariate analysis

Ischaemic and haemorrhagic stroke shared common risk factors (age ≥65 years, CAD, vascular disease, hypertension, diabetes mellitus and sex, all p <0.05), but the weights of these risk factors were different. For example, age ≥65 contributed more to the ischaemic stroke than haemorrhagic stroke (HR, (95%CI), 3.86 (3.72–4.00) for ischemic stroke; 2.88 (2.11–2.46) for haemorrhagic stroke) (Table 2).

**Table 2:** Univariate and multivariate analysis of stroke predictors amongst 425901 Chinese subjects.

	Univariate analysis				Multivariate analysis			
	HR	95%CI		P	HR	95%CI		P
		Lower limit	Higher limit			Lower limit	Higher limit	
**Ischaemic stroke (n = 13274)**								
**Age ≥ 65**	3.98	3.84	4.12	**<0.001**	3.86	3.72	4.00	**<0.001**
*CAD	1.22	1.11	1.36	**<0.001**	1.75	1.58	1.93	**<0.001**
**Vascular disease**	1.81	1.62	2.02	**<0.001**	1.69	1.51	1.89	**<0.001**
**Hypertension**	1.88	1.81	1.97	**<0.001**	1.56	1.49	1.63	**<0.001**
**Diabetes mellitus**	1.22	1.12	1.33	**<0.001**	1.33	1.22	1.45	**<0.001**
*Sex	1.29	1.25	1.34	**<0.001**	1.07	1.03	1.11	**0.001**
*AF	1.69	1.31	2.19	**<0.001**	1.01	0.78	1.32	0.930
Heart failure	1.28	0.86	1.91	0.231	0.86	0.57	1.29	0.459
Renal dysfunction	1.15	0.86	1.53	0.355	0.89	0.66	1.19	0.420
**Haemorrhagic stroke (n = 2917)**								
*CAD	5.29	3.40	8.20	**<0.001**	6.45	4.17	10.20	**<0.001**
*AF	3.75	0.94	14.93	0.062	4.76	1.86	18.87	**0.028**
**Diabetes mellitus**	3.11	2.34	4.12	**<0.001**	3.24	2.44	4.29	**<0.001**
**Vascular disease**	2.06	1.64	2.56	**<0.001**	2.00	1.60	2.51	**<0.001**
**Age ≥ 65**	2.33	2.16	2.50	**<0.001**	2.28	2.11	2.46	**<0.001**
*Sex	1.55	1.43	1.68	**<0.001**	1.39	1.28	1.51	**<0.001**
**Hypertension**	1.29	1.16	1.43	**<0.001**	1.19	1.07	1.32	**<0.001**
Heart failure	0.96	0.36	2.57	0.942	0.99	0.37	2.64	0.984
Renal dysfunction	0.68	0.30	1.51	0.341	0.56	0.25	1.25	0.158

* CAD: coronary artery disease, AF: atrial fibrillation. Sex: male.

In subjects age<75 years, CAD (7.17, 4.14–12.37) and diabetes mellitus (3.27, 2.42–4.42) contributed most to the developing of haemorrhagic stroke than ischaemic stroke (all p<0.001). Amongst the very elderly, vascular disease (2.24, 1.49–3.37) was an additional major risk factor of the haemorrhagic stroke, together with CAD and diabetes mellitus(all p<0.001) (Table 3).

**Table 3:** Multivariate analysis of risk factors for ischaemic and haemorrhagic stroke associated with age strata amongst 425,901 Chinese subjects.

	Age < 75 years(n = 370,414)				Age ≥ 75 years(n = 55,487)			
	HR	95%CI		P	HR	95%CI		P
		Lower limit	Higher limit			Lower limit	Higher limit	
**Ischaemic stroke (n = 13274)**								
*CAD	1.94	1.70	2.22	**<0.001**	1.95	1.66	2.28	**<0.001**
**Diabetes mellitus**	1.44	1.31	1.59	**<0.001**	1.36	1.15	1.61	**<0.001**
**Vascular disease**	1.66	1.44	1.89	**<0.001**	0.85	0.61	1.19	0.343
**Hypertension**	1.47	1.41	1.56	**<0.001**	1.30	1.20	1.41	**<0.001**
*Sex	1.16	1.11	1.22	**<0.001**	1.41	1.33	1.51	**<0.001**
**Age**	1.08	1.07	1.08	**<0.001**	1.01	1.01	1.02	**0.001**
Heart failure	1.47	0.79	2.73	0.225	1.08	0.63	1.82	0.787
Renal dysfunction	1.48	0.99	2.21	0.056	0.90	0.59	1.38	0.639
*AF	1.38	0.90	2.11	0.144	1.51	1.23	1.85	**<0.001**
**Haemorrhagic stroke (n = 2917)**								
*CAD	7.17	4.15	12.37	**<0.001**	7.28	3.46	15.34	**<0.001**
**Diabetes mellitus**	3.27	2.42	4.42	**<0.001**	4.52	2.14	9.52	**<0.001**
**Vascular disease**	1.89	1.44	2.50	**<0.001**	2.24	1.49	3.37	**<0.001**
**Hypertension**	1.19	1.06	1.35	**0.004**	1.23	0.99	1.53	0.060
*Sex	1.61	1.49	1.78	**<0.001**	1.52	1.30	1.78	**<0.001**
**Age**	1.05	1.04	1.05	**<0.001**	1.01	0.99	1.03	0.478
Heart failure	1.29	0.32	5.17	0.717	0.96	0.24	3.85	0.953
Renal dysfunction	1.37	0.62	3.06	0.440	**-**	**-**	**-**	**-**
*AF	2.82	0.70	11.27	0.146	**-**	**-**	**-**	**-**

* CAD: coronary artery disease, AF: atrial fibrillation. Sex: male.

Among population age over 75 years in this cohort, there was no haemorrhagic strokes in the subjects with renal dysfunction (n = 284) and with AF (n = 365).

## Discussion

In this study, our principal findings are as follows: (1) The rate of ischaemic stroke decreased between 2002–2007, then remained broadly similar between 2008–2012, with a similar trend for haemorrhagic stroke; (2) Compared to patients age<65, ischaemic and haemorrhagic stroke incidences were higher in the elderly population, with no significant difference in haemorrhagic stroke between the elderly and the very elderly population (age 65–74 versus age ≥75); and (3) Ischaemic and haemorrhagic stroke shared similar risk factors (age, hypertension, CAD, vascular disease, and diabetes mellitus) in this Chinese population, but the relative weights of risk factors were different.

The changing rates of ischaemic and haemorrhagic stroke were accompanied by a rise in antiplatelet therapy use between 2008 to 2012. Whether this contributed to the decline of stroke rates was uncertain, but increased public awareness and risk factor management, as well as healthcare campaigns, including smoking cessation that could have improved outcomes overall. Nonetheless, stroke rates are still higher than the reported rates in the Caucasian population, confirming the heavy burden of stroke in China. Indeed, the reported stroke deaths in China accounted for 29.4% of total stroke deaths in the world in 2010, although the age-standardized stroke mortality has apparently reduced by 23.9% from 1990 to 2010 [14].

The global burden of stroke is increasing, but globally most of the burden of ischaemic and haemorrhagic stroke is in developing countries, which bear 63% of incident ischaemic strokes and 80% of haemorrhagic strokes [7]. In developing countries, the reported age-adjusted incidence of stroke was 52 per 100000 person-years between 1970–1979 and 117 per 100000 person years in 2000–2008, compared to 63 to 94 per 100 000 person-years in developed countries [8]. The age-adjusted incident first stroke (per 1000 person-years) was reported as 7.6, 6.2, and 5.3 in men, while was 6.2, 5.8, and 5.1 in women in 1950 to 1977, 1978 to 1989, and 1990 to 2004, respectively [15]. The incidence of ICH also decreased between 2000 to 2010, from an annual incidence rate of 5.21/10 000 [95% CI, 4.36–6.24] to 4.30/10 000 [95% CI, 3.21–5.76]) [16].

It is perhaps unsurprising that the risk of ischaemic and haemorrhagic stroke increased with ageing [17,18,19]. The cumulative effects of ageing on the cardiovascular risk factors over a prolonged period would increase stroke risk [3]. However, the precise reasons for the increasing relative rate ratio of ischaemic to haemorrhagic stroke with at age ≥65 in our population are unclear, but could be impacted by the drug therapies. With ageing, for example, the use of antihypertensive drugs (CCB, ACE/ARB), statin, and antiplatelet therapy use was reduced amongst the population with ischaemic stroke. Of note, the rate ratio between age ≥75 years and age 65–74 was broadly comparable. In the Fushimi AF Registry, the most elderly (age ≥85) patients also showed a higher incidence of stroke but similar major bleeding (haemorrhagic stroke, etc.) risks compared with the “younger” AF population (age 75–84 years) [20]. In the Loire Valley atrial fibrillation project, the relative risks of major bleeding also did not increase amongst elderly patients with ageing [21]. The disability and mortality associated with subtypes of stroke could also be related to ethnicity [22,23].

In this Chinese population, common cardiovascular risk factors such as age ≥65 years, hypertension, CAD, diabetes mellitus, and vascular disease, all independently predicted the risk for ischaemic and haemorrhagic stroke, broadly similar to that seen in Western populations [24,25]. We have previously shown that the CHA_2_DS_2_-VASc scores (which is used as a predictor of stroke in AF population), which clusters the common comorbidities, was predictive of ischaemic stroke risk in this large Chinese population [3]. The predictive ability of CHA_2_DS_2_-VASc scores for stroke and thromboembolism has been confirmed in several non-AF population studies [26,27,28].

Unsurprisingly, age-specific clinical risk factors for ischaemic and haemorrhagic stroke could have ethnic differences [8]. In this present Chinese cohort, the average age of population with ischaemic and haemorrhagic stroke was 7 to 8 years younger between 2002 to 2012. The high stroke burden generated by those age<75 years is evident [7]. Both the young and elderly population share common cardiovascular risk factors, but the relative weights of these risk factors could be different. In people age <75 years, CAD and diabetes mellitus contributed more to the developing of haemorrhagic stroke than ischaemic stroke. Amongst the very elderly, vascular disease was another major risk factor for haemorrhagic stroke, together with CAD and diabetes mellitus. In another study, the younger Mexican American population (age 45–59 years) with ischaemic stroke was more likely to have hypertension and diabetes, but less AF compared to non-Hispanic whites [29]. In the INTERSTROKE study, five risk factors were also identified for the risk of ischaemic and intracerebral haemorrhagic stroke, including hypertension, current smoking, abdominal obesity, diet, and physical activity [30]. Indeed, when considering the common clinical cardiovascular risk factors for ischaemic and haemorrhagic stroke, it may be difficult to differentially weigh the risks for ischaemic and haemorrhagic stroke, especially in a population at high risk for both subtypes of stroke. Genetic testing or new biomarkers may be promising future tools to risk stratify for stroke [31]. To take the age-related preventive strategy and to set up more sensitive tools to risk stratify for ischaemic and haemorrhagic stroke could be helpful for reducing the global incidence of stroke.

### Limitations

The major limitation of this study pertains to the use of a medical Insurance administrative dataset. The prevalence of risk factors reported was lower than the hospital-based data, with possible under-reporting and coding errors. However, the high accuracy of ICD9, ICD10 for stroke with administrative datasets have been demonstrated in previous studies [32,33]. The consistency of diagnosis and ICD codes using Chinese Medical Insurance database has been confirmed in our previous study [13]. Although the diagnosis of stroke was confirmed by CT or MRI scanning, data on the severity and disability associated with subtypes of stroke was lacking. Furthermore, there was no data on smoking which was a limitation of this medical insurance dataset. Finally, we used the medical insurance databases affiliated with the Chinese medical insurance scheme in Yunnan Province in the present study, which may have issues on generalizability to across China and other Asian populations.

## Conclusion

In this large Chinese cohort, there was an increased risk of ischaemic stroke compared to haemorrhagic stroke with ageing, which could be associated with different relative weights of risk factors, respectively. CAD and diabetes mellitus contributed more to the developing of haemorrhagic stroke than ischaemic stroke in those age<75 years, whilst vascular disease was an additional major risk factor contributing to haemorrhagic stroke in the very elderly. It highlights the preventive strategy -related to age from ischaemic and haemorrhagic stroke.

## Supporting Information

S1 Checklist (DOCX)

S1 Fig Flowing chart of study population. (TIF)

S1 Table Comorbidity ICD codes. (DOCX)

S2 Table Definitions of comorbidities. (DOCX)
