# Supplementary material for: Determinants and Time Trends for Ischaemic and Haemorrhagic Stroke in a Large Chinese Population
Source: PLoS One. 2016 Sep 29;11(9):e0163171. doi: 10.1371/journal.pone.0163171 (PMC5042494; doi:10.1371/journal.pone.0163171)
Supplement: S1 Checklist — (DOCX) [file pone.0163171.s001.docx]

STROBE Statement—checklist of items that should be included in reports of observational studies

|  | Item No. | Recommendation | Page  No. | Relevant text from manuscript |
| --- | --- | --- | --- | --- |
| **Title and abstract** | 1 | (*a*) Indicate the study’s design with a commonly used term in the title or the abstract | 1 | Determinants and time trends for ischaemic and haemorrhagic stroke in a ‘real world’ Chinese population |
|  |  | (*b*) Provide in the abstract an informative and balanced summary of what was done and what was found | 2 | In this large ‘real world’ Chinese cohort, there was an increased risk of ischaemic stroke compared to haemorrhagic stroke with ageing. CAD, vascular disease, diabetes mellitus, and hypertension were major contributors to the development of hemorrhagic stroke in the very elderly Chinese population. |
| Introduction | | | |  |
| Background/rationale | 2 | Explain the scientific background and rationale for the investigation being reported | 4 | The clinical epidemiology of stroke has been widely investigated in Caucasian populations, but the changes over time in the proportion of ischaemic to haemorrhagic strokes is less clear, especially in developing countries from Asia. |
| Objectives | 3 | State specific objectives, including any prespecified hypotheses | 5 | Our objective was to study the determinants, time trends and relation to age, for first-ever ischaemic and haemorrhagic stroke, in a large ‘real world’ Chinese population cohort over a 10-year observational period. |
| Methods | | | |  |
| Study design | 4 | Present key elements of study design early in the paper | 6 | Databases used in this study have previously been described in detail [12].  The certified validated records provided by the hospitals were included into this governmental medical insurance claims database. Every individual participating in the medical health plan has a permanent and personal registration number, through which every medical ‘event’ could be identified, no matter whether the events happened in clinics and hospitals, and this would be written in the electronic medical records. Data captured included information on demography, diagnosis and treatment of various medical conditions. |
| Setting | 5 | Describe the setting, locations, and relevant dates, including periods of recruitment, exposure, follow-up, and data collection | 6,7,8 | Data for first hospitalization for ischaemic and haemorrhagic strokes from 2002 to 2012 year were retrieved from the medical insurance databases. This study did not include data for outpatients. |
| Participants | 6 | (*a*) *Cohort study*—Give the eligibility criteria, and the sources and methods of selection of participants. Describe methods of follow-up  *Case-control study*—Give the eligibility criteria, and the sources and methods of case ascertainment and control selection. Give the rationale for the choice of cases and controls  *Cross-sectional study*—Give the eligibility criteria, and the sources and methods of selection of participants | 6,7 | A five percent sample was randomly selected from more than 10 million individuals entered into the medical insurance program for the years 2001-2012. Thus, a total of 1,228,639 persons were selected, but after excluding persons with incomplete data (n=2611) and readmissions (n=754,582), we identified 471,446 cases for analysis. Of this cohort, 425,901 without history of stroke were identified. The incidences of ischaemic or haemorrhagic stroke were studied from 2002 to 2012. Over the total follow-up of 1,895,447 person-years, there were 13274 incident ischaemic strokes and 2917 incident haemorrhagic strokes for the final analysis (S1 Fig). |
|  |  | (*b*) *Cohort study*—For matched studies, give matching criteria and number of exposed and unexposed  *Case-control study*—For matched studies, give matching criteria and the number of controls per case | NA | NA |
| Variables | 7 | Clearly define all outcomes, exposures, predictors, potential confounders, and effect modifiers. Give diagnostic criteria, if applicable | 7,8 | Evaluation of ischaemic stroke, haemorrhagic stroke and comorbidities  ICD-9, ICD-10 codes defined cardiovascular disease and other comorbidities are shown in S1 Table. The definition of various comorbidities is summarized in S2 Table. |
| Data sources/ measurement | 8* | For each variable of interest, give sources of data and details of methods of assessment (measurement). Describe comparability of assessment methods if there is more than one group | 6 | The Chinese National Health Insurance program, which includes the Chinese medical insurance scheme, and Rural Cooperative Medical System, provide the basic medical care to urban and rural residents. The Chinese medical insurance scheme was started in December, 1998, and this program provides coverage for inpatient and outpatient medical services to approximately 597 million Chinese urban residents in 2014 (and covers 95% of total urban and rural residents in China). The local government maintains identical electronic clinical information on all health care provided to insured patients from the different provinces in China. In brief, we used the medical insurance databases affiliated with the Chinese medical insurance scheme in Yunnan Province, China, from January 1, 2001 through December 30, 2012. |
| Bias | 9 | Describe any efforts to address potential sources of bias | 15 | The major limitation of this study pertains to the use of a medical Insurance administrative dataset. The prevalence of risk factors reported was lower than the hospital-based data, with possible under-reporting and coding errors. |
| Study size | 10 | Explain how the study size was arrived at | 6,7 | A five percent sample was randomly selected from more than 10 million individuals entered into the medical insurance program for the years 2001-2012. Thus, a total of 1,228,639 persons were selected, but after excluding persons with incomplete data (n=2611) and readmissions (n=754,582), we identified 471,446 cases for analysis. Of this cohort, 425,901 without history of stroke were identified. The incidences of ischaemic or haemorrhagic stroke were studied from 2002 to 2012. Over the total follow-up of 1,895,447 person-years, there were 13274 incident ischaemic strokes and 2917 incident haemorrhagic strokes for the final analysis (S1 Fig). |

Continued on next page

| Quantitative variables | 11 | Explain how quantitative variables were handled in the analyses. If applicable, describe which groupings were chosen and why | 8 | Incident ischaemic and haemorrhagic strokes (per 1000 person-years, 95% confidential interval, CI) were calculated in this population during a 10-year period. The rates of ischaemic and haemorrhagic stroke were calculated in relation to different age group categories (age <65 years, age 65-74 years, and age ≥75 years), and the relative rate ratio for ischaemic to haemorrhagic stroke in the three age groups over time were compared. |
| --- | --- | --- | --- | --- |
| Statistical methods | 12 | (*a*) Describe all statistical methods, including those used to control for confounding | 8,9 | Continuous variables were tested for normality by the Kolmogorov-Smirnov test. Those with a normal distribution are presented as a mean (standard deviation, SD) and analyzed using t test. Data with a non-normal distribution are presented as median (inter-quartile range, IQR). The comparison of discrete variables was performed using the chi-square test. |
|  |  | (*b*) Describe any methods used to examine subgroups and interactions | 9 | A multivariate analysis was used to determine cardiovascular risk factors predicting the occurrence of ischaemic and haemorrhagic stroke in the general populations, respectively. Factors associated with stroke were included into the Cox hazard proportional models, including age≥65, sex, CAD, vascular disease, hypertension, diabetes mellitus, atrial fibrillation (AF), heart failure(HF), and renal dysfunction. Hazard ratios (HR) of cardiovascular risk factors for ischaemic and haemorrhagic stroke were estimated by a Cox proportional hazards model. |
|  |  | (*c*) Explain how missing data were addressed | 7 | Thus, a total of 1,228,639 persons were selected, but after excluding persons with incomplete data (n=2611) and readmissions (n=754,582), we identified 471,446 cases for analysis. |
|  |  | (*d*) *Cohort study*—If applicable, explain how loss to follow-up was addressed  *Case-control study*—If applicable, explain how matching of cases and controls was addressed  *Cross-sectional study*—If applicable, describe analytical methods taking account of sampling strategy | 15 | The prevalence of risk factors reported was lower than the hospital-based data, with possible under-reporting and coding errors. However, the high accuracy of ICD9, ICD10 for stroke with administrative datasets have been demonstrated in previous studies [30,31]. |
|  |  | (*e*) Describe any sensitivity analyses | 15 | The consistency of diagnosis and ICD codes using Chinese Medical Insurance database has been confirmed in our previous study [12]. |
| Results | | | | |
| Participants | 13* | (a) Report numbers of individuals at each stage of study—eg numbers potentially eligible, examined for eligibility, confirmed eligible, included in the study, completing follow-up, and analysed | 10 | Among 425,901 individuals without prior stroke (52.4% male, median age 54), there were 13274 (63.8% male, median age 69) incident ischaemic strokes between 2002 to 2012. |
|  |  | (b) Give reasons for non-participation at each stage | NA | NA |
|  |  | (c) Consider use of a flow diagram | 7 | Over the total follow-up of 1,895,447 person-years, there were 13274 incident ischaemic strokes and 2917 incident haemorrhagic strokes for the final analysis (S1 Fig). |
| Descriptive data | 14* | (a) Give characteristics of study participants (eg demographic, clinical, social) and information on exposures and potential confounders | 10 | Hypertension, diabetes mellitus and CAD were the most comorbidities (Table 1). |
|  |  | (b) Indicate number of participants with missing data for each variable of interest |  |  |
|  |  | (c) *Cohort study*—Summarise follow-up time (eg, average and total amount) | 7 | Over the total follow-up of 1,895,447 person-years, there were 13274 incident ischaemic strokes and 2917 incident haemorrhagic strokes for the final analysis (S1 Fig). |
| Outcome data | 15* | *Cohort study*—Report numbers of outcome events or summary measures over time | 10 | Ischaemic stroke rates (per 1000 patient-years, 95% CI) were 6.99 (6.90-7.10) during the 10-year period. The rate of ischaemic stroke decreased between 2002-2007, then remained broadly similar between 2008-2012. |
|  |  | *Case-control study—*Report numbers in each exposure category, or summary measures of exposure | NA |  |
|  |  | *Cross-sectional study—*Report numbers of outcome events or summary measures | NA |  |
| Main results | 16 | (*a*) Give unadjusted estimates and, if applicable, confounder-adjusted estimates and their precision (eg, 95% confidence interval). Make clear which confounders were adjusted for and why they were included | 11 | Ischaemic and haemorrhagic stroke shared common risk factors (age ≥65 years, CAD, vascular disease, hypertension, diabetes mellitus and sex, all p <0.05), but the weights of these risk factors were different. For example, age ≥65 contributed more to the ischaemic stroke than haemorrhagic stroke (HR, (95%CI), 3.86 (3.72-4.00) for ischemic stroke; 2.88 (2.11-2.46) for haemorrhagic stroke) (Table 2). |
|  |  | (*b*) Report category boundaries when continuous variables were categorized | 8 | The rates of ischaemic and haemorrhagic stroke were calculated in relation to different age group categories (age <65 years, age 65-74 years, and age ≥75 years), and the relative rate ratio for ischaemic to haemorrhagic stroke in the three age groups over time were compared. |
|  |  | (*c*) If relevant, consider translating estimates of relative risk into absolute risk for a meaningful time period | NA | NA |

Continued on next page

| Other analyses | 17 | Report other analyses done—eg analyses of subgroups and interactions, and sensitivity analyses | 12 | In subjects age<75 years, CAD (7.17, 4.14-12.37) and diabetes mellitus (3.27, 2.42-4.42) contributed most to the developing of haemorrhagic stroke than ischaemic stroke (all p<0.001). Amongst the very elderly, vascular disease (2.24, 1.49-3.37) was an additional major risk factor of the haemorrhagic stroke, together with CAD and diabetes mellitus(all p<0.001) (Table 3). |
| --- | --- | --- | --- | --- |
| Discussion | | | | |
| Key results | 18 | Summarise key results with reference to study objectives | 13 | In this study, our principal findings are as follows: (1) The rate of ischaemic stroke decreased between 2002-2007, then remained broadly similar between 2008-2012, with a similar trend for haemorrhagic stroke; (2) Compared to patients age<65, ischaemic and haemorrhagic stroke incidences were higher in the elderly population, with no significant difference in haemorrhagic stroke between the elderly and the very elderly population (age 65-74 versus age ≥75); and (3) Ischaemic and haemorrhagic stroke shared similar risk factors (age, hypertension, CAD, vascular disease, and diabetes mellitus) in this Chinese population, but the relative weights of risk factors were different. |
| Limitations | 19 | Discuss limitations of the study, taking into account sources of potential bias or imprecision. Discuss both direction and magnitude of any potential bias | 15,16 | The major limitation of this study pertains to the use of a medical Insurance administrative dataset. The prevalence of risk factors reported was lower than the hospital-based data, with possible under-reporting and coding errors. However, the high accuracy of ICD9, ICD10 for stroke with administrative datasets have been demonstrated in previous studies [30,31]. The consistency of diagnosis and ICD codes using Chinese Medical Insurance database has been confirmed in our previous study [12]. Although the diagnosis of stroke was confirmed by CT or MRI scanning, data on the severity and disability associated with subtypes of stroke was lacking. Finally, there was no data on smoking which was a limitation of this medical insurance dataset. |
| Interpretation | 20 | Give a cautious overall interpretation of results considering objectives, limitations, multiplicity of analyses, results from similar studies, and other relevant evidence | 13,14,15,16 | In this large ‘real world’ Chinese cohort, there was an increased risk of ischaemic stroke compared to haemorrhagic stroke with ageing. CAD and diabetes mellitus contributed more to the developing of haemorrhagic stroke than ischaemic stroke in those age<75 years, whilst vascular disease was an additional major risk factor contributing to haemorrhagic stroke in the very elderly. |
| Generalisability | 21 | Discuss the generalisability (external validity) of the study results | 16 | Finally, we used the medical insurance databases affiliated with the Chinese medical insurance scheme in Yunnan Province in the present study, which may have issues on generalizability to across China. |
| Other information | |  | | |
| Funding | 22 | Give the source of funding and the role of the funders for the present study and, if applicable, for the original study on which the present article is based | 1 | The study was supported by Chinese PLA Healthcare Foundation (13BJZ40), Beijing Natural Science Foundation (7142149), and National Natural Science Foundation of China (H2501). |

*Give information separately for cases and controls in case-control studies and, if applicable, for exposed and unexposed groups in cohort and cross-sectional studies.

**Note:** An Explanation and Elaboration article discusses each checklist item and gives methodological background and published examples of transparent reporting. The STROBE checklist is best used in conjunction with this article (freely available on the Web sites of PLoS Medicine at http://www.plosmedicine.org/, Annals of Internal Medicine at http://www.annals.org/, and Epidemiology at http://www.epidem.com/). Information on the STROBE Initiative is available at www.strobe-statement.org.
