# Supplementary figures and images for: Determinants and Time Trends for Ischaemic and Haemorrhagic Stroke in a Large Chinese Population
Source: PLoS One. 2016 Sep 29;11(9):e0163171. doi: 10.1371/journal.pone.0163171 (PMC5042494; doi:10.1371/journal.pone.0163171)

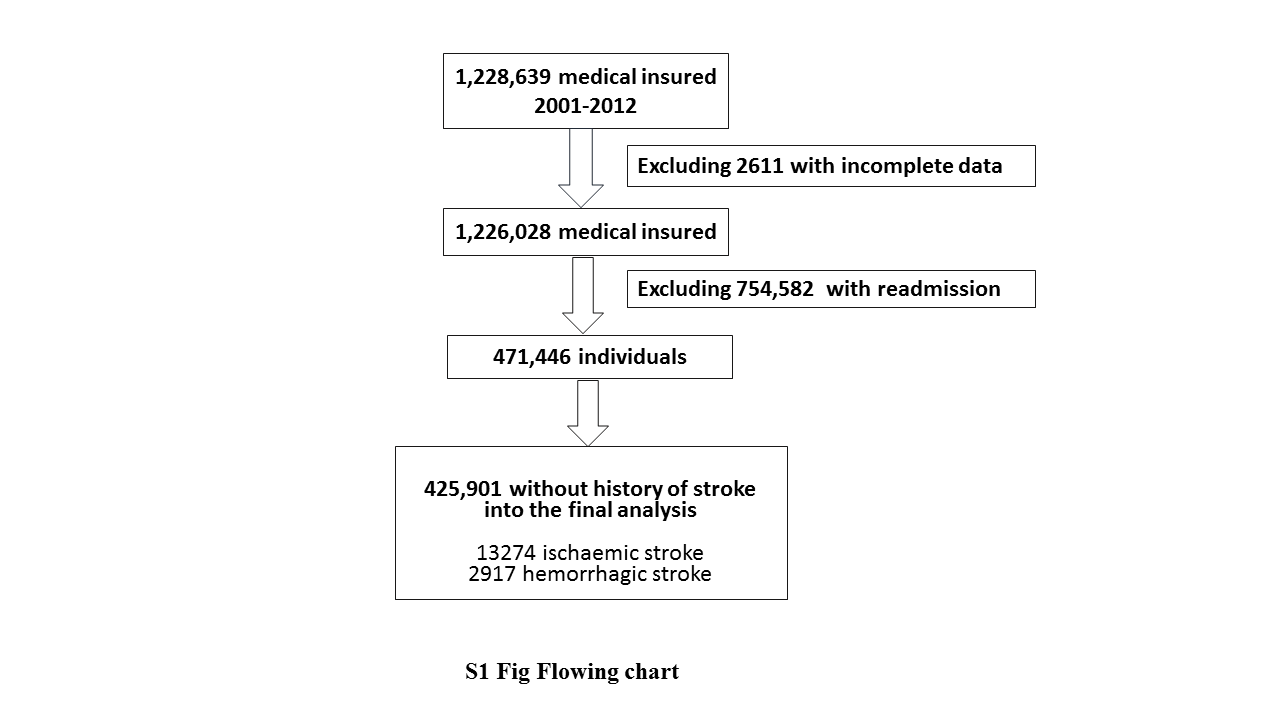

Supplement: S1 Fig — (TIF) [file pone.0163171.s002.tif]
