# Supplementary material for: Determinants and Time Trends for Ischaemic and Haemorrhagic Stroke in a Large Chinese Population
Source: PLoS One. 2016 Sep 29;11(9):e0163171. doi: 10.1371/journal.pone.0163171 (PMC5042494; doi:10.1371/journal.pone.0163171)
Supplement: S1 Table — (DOCX) [file pone.0163171.s003.docx]

**S1 Table. Comorbidity ICD codes**

| Comorbidity | ICD-9 codes | ICD-10 codes |
| --- | --- | --- |
| Rheumatic heart disease | 393–398 | I05,I06,I07,I09.9 |
| Heart failure | [428](http://www.icd9data.com/getICD9Code.ashx?icd9=428) | I42, I50, I110,J819 |
| Dilated cardiomyopathy | 425.4 | I42.0 |
| Diabetes | 249–250 | E10-E14 |
| Hypertension | 401–405 | I10-I15 |
| Coronary artery disease | 410-414 | I20-I25 |
| Myocardial infarction | 410 | I21, I22 |
| Peripheral vascular disease | 440.2 | I65, I70-74 |
| Ischemic stroke | 436 | I63 |
| Hemorrhagic stroke | 430,431,432 | I60.x,I61.x |
| Hemoptysis | 786.3 | R04.2 |
| Gastrointestinal bleeding | R04.201, J47.X02 | K92.208,K92.204,K92.207, K27.404 |
| Chronic obstructive pulmonary disease | 490–496 | J42,J44.0-9 |
| Hyperlipidemia | 272.4 | E78.0-3,E78.5 |
| Renal dysfunction | 585,586 | M1A.3 |
| Hyperthyroidism | 242 | E05 |
| Hypothyroidism | 244 | E03 |
| Atrial fibrillation | [427.31](http://www.icd9data.com/getICD9Code.ashx?icd9=427.31) | I48 |
