# Supplementary material for: Determinants and Time Trends for Ischaemic and Haemorrhagic Stroke in a Large Chinese Population
Source: PLoS One. 2016 Sep 29;11(9):e0163171. doi: 10.1371/journal.pone.0163171 (PMC5042494; doi:10.1371/journal.pone.0163171)
Supplement: S2 Table — (DOCX) [file pone.0163171.s004.docx]

**S2 Table. Definitions of comorbidities**

| Comorbidity | Definition |
| --- | --- |
| Hypertension | A resting blood pressure ≥ 140mmHg systolic and/or ≥90 mmHg diastolic on at least 2 occasions or current antihypertensive drug treatment |
| Coronary artery disease | Prior myocardial infarction, angina pectoris, percutaneous coronary intervention or coronary artery bypass surgery |
| Heart failure | The presence of signs and symptoms of either right (elevated central venous pressure, hepatomegaly, dependent edema) or left ventricular failure (exertional dyspnea, cough, fatigue, orthopnea, paroxysmal nocturnal dyspnea, cardiac enlargement, rales, gallop rhythm, pulmonary venous congestion) or both, confirmed by non-invasive or invasive measurements demonstrating objective evidence of cardiac dysfunction |
| Peripheral vascular disease | Intermittent claudication, previous surgery or percutaneous intervention on the abdominal aorta or the lower extremity vessels, abdominal or thoracic surgery, arterial and venous thrombosis |
| Diabetes | Fasting plasma glucose concentration ≥ 7.0 mmol/l, or two hour plasma glucose concentration ≥ 11.1 mmol/l after 75g anhydrous glucose in an oral glucose tolerance test |
| Rheumatic heart disease | Damaged heart valves that can be a narrowing or leakage, particularly mitral valve (mitral valve stenosis) as a result of residual symptoms of rheumatic fever |
| Dilated cardiomyopathy | The heart becomes weakened and enlarged, and it cannot pump blood efficiently. |
| Renal dysfunction | The presence of chronic dialysis, renal transplantation, or an estimated glomerular filtration rate (eGFR) <60 mL/min per 1.73 m^2^ |
| Liver dysfunction | The presence of liver disease with serum levels of either ALT (SGPT), AST (SGOT), or alkaline phosphatase above 3 x upper limit of normal (ULN) |
| Hyperlipidemia | An elevation of [lipids](http://cholesterol.about.com/od/aboutcholesterol/g/lipid.htm) or [lipoproteins](http://www.news-medical.net/health/What-are-Lipoproteins.aspx) in the blood, e.g. fasting triglyceride level >200 mg/dL, LDL-L>160mg/dL , and non-HDL-C>190mg/dl |
| Hyperthyroidism | High levels of thyroxine and low or nonexistent amounts of thyroid stimulating hormone (TSH) |
| Hypothyroidism | Low thyroxine and high TSH levels |
| Chronic obstructive pulmonary disease | A nonreversible lung disease that is a combination of emphysema and chronic bronchitis |
| Ischemic stroke | A focal neurologic deficit of sudden onset diagnosed clinically by a neurologist based on CT or MRI scanning |
